# Harnessing Marine Biocatalytic Reservoirs for Green Chemistry Applications through Metagenomic Technologies

**DOI:** 10.3390/md16070227

**Published:** 2018-07-04

**Authors:** Ignacio Abreu Castilla, David F. Woods, F. Jerry Reen, Fergal O’Gara

**Affiliations:** 1 BIOMERIT Research Centre, School of Microbiology, University College Cork, T12 K8AF Cork, Ireland; ignacio.abreucastilla@ucc.ie (I.A.C.); david.woods@ucc.ie (D.F.W.); 2 School of Microbiology, University College Cork, T12 K8AF Cork, Ireland; j.reen@ucc.ie; 3 Telethon Kids Institute, Perth, WA 6008, Australia; 4 Human Microbiome Programme, School of Pharmacy and Biomedical Sciences, Curtin Health Innovation Research Institute, Curtin University, Perth, WA 6102, Australia

**Keywords:** green chemistry, chemical industries, biocatalysis, metagenomics, marine, biodiscovery, lipase, esterase

## Abstract

In a demanding commercial world, large-scale chemical processes have been widely utilised to satisfy consumer related needs. Chemical industries are key to promoting economic growth and meeting the requirements of a sustainable industrialised society. The market need for diverse commodities produced by the chemical industry is rapidly expanding globally. Accompanying this demand is an increased threat to the environment and to human health, due to waste produced by increased industrial production. This increased demand has underscored the necessity to increase reaction efficiencies, in order to reduce costs and increase profits. The discovery of novel biocatalysts is a key method aimed at combating these difficulties. Metagenomic technology, as a tool for uncovering novel biocatalysts, has great potential and applicability and has already delivered many successful achievements. In this review we discuss, recent developments and achievements in the field of biocatalysis. We highlight how green chemistry principles through the application of biocatalysis, can be successfully promoted and implemented in various industrial sectors. In addition, we demonstrate how two novel lipases/esterases were mined from the marine environment by metagenomic analysis. Collectively these improvements can result in increased efficiency, decreased energy consumption, reduced waste and cost savings for the chemical industry.

## 1. The Nature of the Modern Chemical Industry

Many chemical processes are time-consuming and have high associated costs. The atom economy of many chemical processes is extremely inefficient, producing up to 100 times more waste and by-products than the desired end product [1]. The Environmental Quotient (EQ) was introduced to highlight this problem and provides a method of analysing reaction efficiency and pollution [2]. Other important methods of reaction evaluation include the Reaction Mass Efficiency (RME) and the Mass Intensity (MI) [3]. The Life-Cycle Assessment (LCA) is a comprehensive approach to measure the environmental impact of a product. This technique measures the impact from the sourcing of the raw material, through any processes involved in its production and usage, as well as any disposal/recycling treatments [4]. Outlined in Table 1 are some of the most important tools and techniques that green chemistry uses to measure environmental impact, effectiveness and efficiency of processes. In addition to processing issues, it is estimated that the chemical industry is responsible for 25% of all of the industrial energy consumption in the U.S. annually [5]. These inefficiencies, coupled with high energy consumption, place a significant financial burden on the industry and need to be addressed.

In addition to challenges associated with reaction conditions and high input costs, the chemical industries produce waste pollutants that can be costly to process and dispose of. Pollution comes about through routine production processes and also through accidental contamination, both of which can be expensive to resolve [6]. Environmental issues related to pollution are high on governmental agendas and are being thoroughly investigated. These include, but are not limited to, air pollution, water treatment, waste monitoring, waste minimization, and process improvement [7,8,9,10]. The majority of the waste and by-products generated by chemical industries are known to be directly toxic to human health [11]. Atmospheric pollution, is now considered the principal health risk to humans and has recently been defined as a public health emergency [12]. Of the world’s population, 92% of people are living with levels of air pollution exceeding the upper safety limits established by the World Health Organisation (WHO) [13]. Total global premature deaths from airborne toxicants are estimated to be in excess of 15 million people annually [14]. Furthermore, indirect effects of pollution can also be detrimental as with aquatic and terrestrial pollutants. Hence, there is recognition for the need to establish strong environmental regulations to assure enhanced wellbeing and a sustainable future for the growing population that inhabits the Earth. For the chemical industry, overheads, process inefficiencies, high costs, and pollution are issues that need to be addressed with haste. Green chemistry provides an apt and apropos solution that can address many of these issues, as well as proving significant added value to existing and new pipeline developments.

### 1.1. The Application and Benefits of Utilising Green Chemistry Solutions 

Green chemistry is broadly defined as the application of 12 principles that lead to chemical products and processes becoming more efficient, less toxic, and producing less waste [15]. These principles can be divided into two broad groupings, (1) principles relating to reducing energy utilisation and waste products and (2) principles relating to safer processes and products. Enzymes have biocatalytic properties and have contributed greatly to the integration of green chemistry solutions into the chemical industry [16]. 

**Figure 1:**
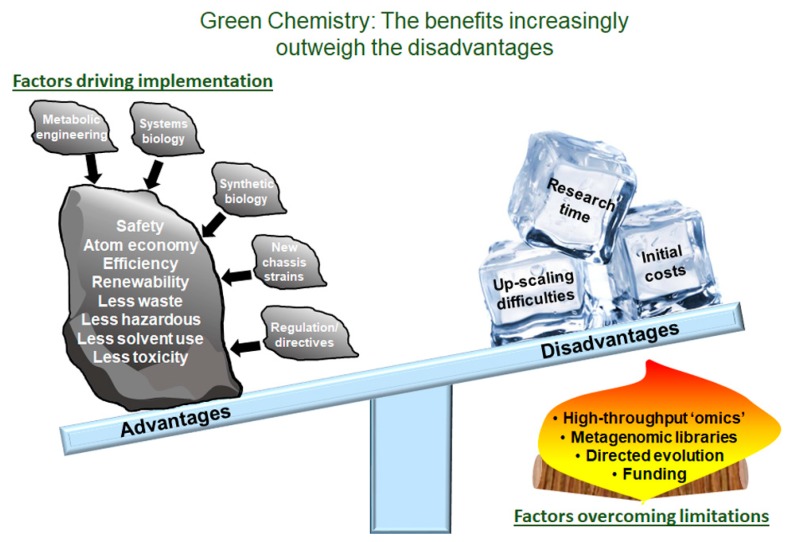
Green Chemistry: The Benefits Increasingly Outweigh the Disadvantages. Overview of the advantages and the disadvantages of green chemistry. Included are several solutions and strategies that combat the current disadvantages. These solutions and strategies include advancements in high-throughput screening, metagenomic library construction, directed evolution and funding opportunities.

The use of biocatalysts has been gaining strong momentum in the last number of years in an attempt to deliver a more environmentally safe sustainable future [17,18,19]. The EU-COST Action D29 is a good example of frameworks set up to establish green chemical solutions. This initiative has shown great success in applying green chemistry to solve a wide array of problems, from reducing reaction temperatures to improving atom economy [20,21]. The chemical industry has also committed to producing more environmentally friendly solutions, where biocatalytic technologies are to the fore [22]. There has been expansion in the application of enzyme biotechnology to the industrial sector. This is mainly due to the environmentally friendly nature of enzymes, their ability to carry out reactions more rapidly and efficiently than conventional processes and the associated reduction in production costs [23,24]. There are now numerous examples of processes using biocatalysts that are vastly more cost effective than their traditional counterparts [25,26,27,28,29,30]. We have outlined the benefits and diminishing limitations of using green chemistry in Figure 1.

Both public and private sectors recognise the advantages in greener manufacturing and are, therefore, heavily investing in projects such as “Chemical Manufacturing Methods for the 21st Century” (CHEM21). This project is part of the EU Innovative Medicines Initiative (IMI) and aims to support a transition towards more environmentally friendly drug manufacture. Six pharmaceutical companies, thirteen European universities, and five small to medium enterprises are currently involved in this initiative [22]. CHEM21 has noted that advancements in biocatalysis are made more feasible with advancements in technologies such as bioinformatics and high-throughput screening. The global economic enzyme market in 2016 was worth over $5 billion growing from $1.5 in 1998 [31,32]. It is estimated that this figure will continue to grow and the industry will be valued in excess of $6.3 billion by 2021 [33]. 

### 1.2. Replacement of Traditional Chemical Processes by Greener Chemical Solutions

Improving the productivity and yield by microbial metabolic networks is imperative in order to replace traditional chemical processes with greener alternatives. This has led to the development of microbial cell factories. Tools such as systems metabolic engineering and synthetic biology have become useful to modify metabolic pathways, control gene expression, and increase the tolerance of microbial cells to chemicals. These tools and the increased knowledge has added new dimensions to the integration of biocatalysis within existing drug synthesis pipelines [34]. Similarly, advances in key technologies has enabled industry to apply microbial biotechnology to produce chemicals, intermediates, and final products, with improved titre, product, and yields. Notwithstanding the early successes, challenges remain. While academic research has gone some way towards elucidating the metabolic parameters within which biocatalysts will need to operate in situ, often these do not translate easily into large scale production systems [35]. Engineering of new chassis organisms for heterologous biocatalytic conversions in small to medium scale reactors may not condition the cell for production in industry-scale bioreactors. Furthermore, issues regarding stability of heterologous DNA, regulation surrounding the use of genetically modified organisms, access to genetic resources as addressed by the Nagoya protocol, and homogeneity of conditions within large scale bioreactors are key considerations that must be met. The ultimate aim is a ‘one-pot’ solutions where several engineered strains combine to deliver a product pipeline, will require intensive technological developments. We will need to further our understanding of microbial-microbial interactions. Dynamic communication within microbial communities is referred to as interactomics. In spite of interactomics challenges, the integration of biocatalytic systems in industrial biotechnology has seen several successes.

**Figure 2:**
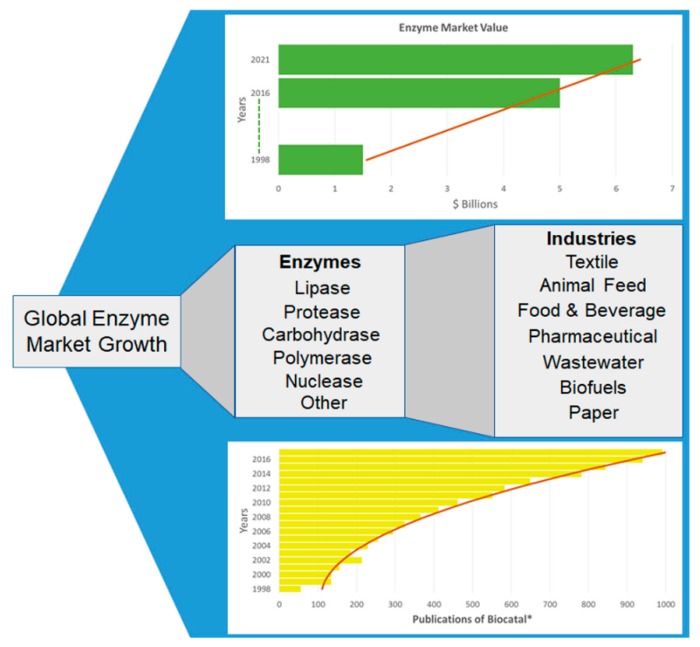
The Growth and Application of the Enzyme Market. Listed are some of the enzyme categories used by industry and the types of industries where the enzymes are widely employed. A PubMed database was searched for the number of publications from 1998 to 2016 in relation to biocatalysis using the following query: (biocatal*[Title/Abstract]). The total value of the enzyme market is also included, from 1998 [32], to 2016 and the onward estimation up until 2021. The calculated trend-lines clearly show an increase in both market value and biocatalysts discovered.

The common enzymes that are applicable to industry are outlined in Figure 2. In the papermaking process, lipases are replacing cleaning agents and talc which were used to avoid the forming of pitch [36], the insoluble material produced during the mechanical processing of pulp [37]. This pitch can lead to a deterioration in the paper quality and also damage machinery used for paper production. Since the introduction of lipases, this process has greatly reduced its energy consumption and environmental impact [38]. Esterases are also used in recycled paper processing replacing highly toxic solvents. These enzymes stop the agglomeration of glues by hydrolysing the polyvinyl acetate, the main component of glues [39]. It is estimated that in the U.S., one billion dollars are lost due to glue agglomeration [40]. Enzymes such as laccase and xylanase are used to bleach paper, these substitute for harmful chlorine and alkali chemicals [41,42]. These enzymes demonstrate practical fulfilment of the principles of green chemistry by increasing atom economy, reducing cost and decreasing waste [38]. 

Microplastics have been highlighted as a global pollutant. A recent review has detailed eighty-three studies identifying microplastics in the marine environment [42]. While concentrations varied, the microplastics were found distributed throughout the world. These microplastics affect the fertility of marine organisms, such as oysters and copepods [43,44]. These particles can then be transferred to humans via the food chain [44]. The plastic polyethylene (PE), is one of the largest manufactured microplastics worldwide and one of the least recycled. Biocatalysis can be used to metabolise this waste plastic to form useful value-added biopolymers [45]. Food and beverage industries are responsible for major environmental issues [46] and with the population of the world predicted to increase significantly the use of enzymes such as cellulases, pectinases, and amylases which are used in clarification and maceration, will allow and facilitate the demand for efficient food and beverage production. The use of these enzymes leads to cost reductions and improvement in the quality of fruit juice [47]. In the bakery sector, amylases and lipases are used to increase softness, flavour, and to prolong the shelf life of products [48]. During the production of cheeses, lipases are often used to enhance the flavour and save on milk consumption [49].

Lipases, combined with other enzymes such as proteases, amylases and cellulases, play an important role in the detergent industry. These enzymes are able to remove stains without releasing toxic substances and can retain activity at low temperatures. The use of these enzymes could eventually replace surfactants [50], which are extremely harmful for aquatic environments [51]. Both amylases and proteases are successfully used in dishwasher detergents to remove carbohydrates and proteins [52]. These enzymes can replace some of the phosphates and bleaching agents that are highly harmful to the environment [53].

The pharmaceutical industry is also seeking alternatives to traditional chemical processes, in order to create new greener processes with decreased hazardous waste and reduced costs. Therefore, the application of biocatalysts has been gaining interest over time in the medical and pharmaceutical industry [23,54]. An example of this is with the antidiabetic drug, Sitagliptin. This drug is traditionally produced using high pressure in conjunction with expensive rhodium catalysts, however biocatalytic technologies replaced this method and improved productivity by 53% as well as reducing waste by 19% [55]. Another practical example is the use of the enzyme keto-reductase which is involved in the production of the anti-asthmatic drug, Singulair. The use of this keto-reductase produces a very pure (>99.9% ee; this is a measurement of the production of the desired enantiomer) and high yields (>95%) of the drug. Moreover this enzyme replaces the hazardous molecule (-)-B-chlorodiisopinocampheylborane which was used in the chemical production of the drug [56]. Artemisinin, a sesquiterpene lactone, was originally isolated from the sweet wormwood plant (*Artemisia annua* L.) [57]. It is used in the treatment of malaria, however, the sweet wormwood plant is very rare and makes the production of the drug expensive [58]. In an effort to increase the production of artemisinin, biocatalysis has been employed in the production of precursors of the drug to decrease time and cost expenditures. A *Saccharomyces cerevisiae* strain was engineered by chromosomal integration to produce high levels of the artemisinin precursor, artemisinic acid. Using this engineered yeast in the production of artemisinin would be relatively easy, environmentally friendly, and cost effective due to reduced contamination and solvents needed when compared the plant extract techniques [59]. 

**Figure 3:**
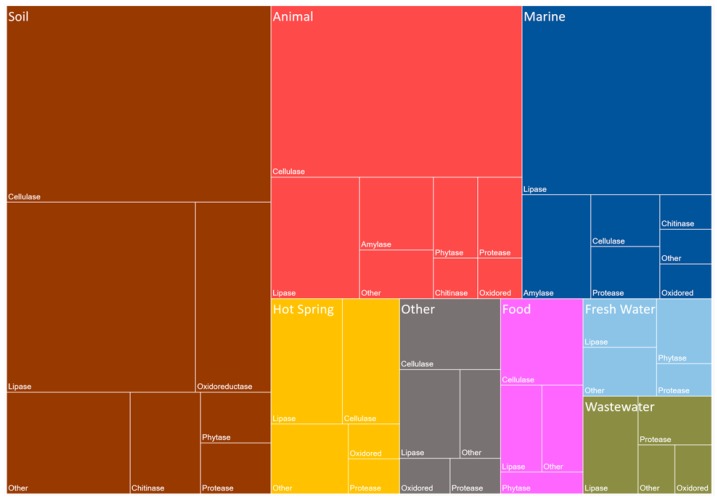
Industrially Relevant Enzymes Discovered by Metagenomics and Grouped by their Sources. The published industrially important enzymes between the years 2014 and 2018 were compiled. Each of the publications found between 2014/01/01 and 2018/04/04 were manually checked and only industrially relevant enzymes identified by metagenomic analysis were included. We acknowledge the data collection by Berini et al., 2017 in the aid of compiling this figure [61]. Each of the enzymes were grouped into a class and a general source. The enzymes are grouped into the following enzymatic categories, Amylase, Cellulase, Chitinase, Lipase (including Esterases), Oxidoreductase (synopsised as Oxidored), Phytase (including Phosphatases), Protease and Other. Each of the sources of the metagenomic libraries were grouped into different environments including, Soil, Animal, Marine, Hot Springs, Food, Fresh Water, Waste Water, and Other. The “Other” class and group contained all data points that were not grouped into the defined categories. The graphic is based on the number of enzymes that were sourced from a specific environment.

The discovery of new enzymes that conduct industrially important reactions is vital for the industry’s future progression. The most successful and effective system for the discovery of novel enzymes is with metagenomic technology. A hierarchical diagram, a Treemap, representing defined publications was formed with nested rectangles corresponding to publication abundance. The constructed Treemap (Figure 3) shows the biocatalysts that are industrially relevant and discovered by metagenomic screens between the years 2014 to 2018. The Treemap was created using a PubMed database search (accession on 4 April 2018 with the following query: (metagenome*[Title/Abstract]) AND (X[Title/Abstract]) AND (‘2014/01/01’[Date—Publication]: ‘2018/04/04’[Date—Publication]) (X was substituted for each enzymatic class). Screens of metagenomic libraries of soil samples, have provided the most industrially important enzymes, followed by animal and marine biomes/ecosystems. The most abundant enzymes discovered in the soil and animal sources are cellulases, while lipases/esterases are the most abundant from the marine environment. Lipases/esterases have previously been noted as having one of the most important impacts on chemical processes and is applicable to a wide range of industries such as the food, detergent and pharmaceutical industries [60].

## 2. Resources for the Discovery of Green Chemical Biocatalysts

All plants, animals and microorganisms produce a vast array of enzymes capable of catalysing a myriad of reactions. Biocatalysis has been utilised for thousands of years in the production of food and beverages such as cheeses and wines. While much success has been made with biocatalysts obtained from animals (such as pepsin [62]) and plants (such as peroxidases [63]), microorganisms provide a significant reservoir of enzymes that are easily screened for function on a large scale. Microbial enzymes can be produced in a controlled system and utilise relatively inexpensive growth media. Combined with the ease of sequencing and annotation due to their genome size and complexity, this makes microbes an ideal target for catalytic biodiscovery. 

In the search for novel biocatalysts, the environment that the microorganisms inhabit must be taken into consideration. The marine ecosystem occupies more than 70% of Earth’s surface and offers a wide range of potential biocatalysts that have not previously been investigated. Fossil records have demonstrated that marine bacterial life predated terrestrial bacteria by 600 million years [64]. Only 16% of defined living species are characterised from the marine environment [65], and it is one of the least surveyed environments of any biome present on the planet, with less than 0.0001% of the area of the deep oceans having been explored [66]. However the vast majority of these microorganisms cannot be cultivated in the laboratory by current practices [67]. This is due to many factors, including our inability to reproduce or mimic the nutrient and physicochemical parameters needed to facilitate the growth of these microorganisms. This causes a bottleneck in enzyme biodiscovery, which can now be addressed, in part, by the use of metagenomic tools. 

The varied and extreme conditions of the marine biome, such as pH, low temperature, high salinity, and high-pressure levels result in potentially industrially favourable enzymatic adaptations. Extremophilic bacteria are microorganisms that have adapted to these harsh conditions. These bacteria can produce extremozymes which are enzymes that catalyse reactions under atypical conditions [68]. These enzymes are a good example of enzymatic adaptations [69,70]. This makes these enzymes important to chemical industries as often chemical processes need to be conducted under aberrant conditions [71]. There are several examples of extremozymes with promising industrial value (Table 2) [72,73,74,75,76,77,78,79]. 

Globally, it is estimated that 90% of the biosphere exists at temperatures below 10 °C [80,81]. There is great applicability for cold active enzymes from these environments to the food, beverage and detergent market. Lipases from *Bacillus pumilus* were isolated from the Antarctic and these enzymes maintained 80% of their activity at 10 °C, thus demonstrating the potential industrial application for the food, organic synthesis and detergent industry [75]. Cold active alpha-amylases from *Pseudoalteromonas haloplanktis* have maintained 80% of its activity at high salt concentration [79]. A cold-adapted lipase from *Photobacterium lipolyticum* was discovered and maintained activity at 5 °C [76]. In contrast to cold active enzymes, thermophilic enzymes have also been uncovered that have industrial importance, such as a protease from a *Marinobacter* species, which retains 60% of their activity at 80 °C [77]. 

Sponges from the marine environment are of particular interest as resources for novel microbial enzymes [82]. Marine sponges possess a large number of pores that circulate water and filter nutrients and microorganisms. This develops a niche environment where microbial communities can grow and thrive [83]. Many microbes inhabit these niches within sponges, where often a symbiotic mutualism may occur. The bacteria residing in the sponge, can be nearly half the biomass of the entire sponge making them a rich resource of bacteria [84,85]. This rich resource of bacteria can be mined for potential novel activities. Sponges have successfully been mined for their microbial biocatalyst content with a wide range of enzymes being successfully discovered from a number of different sponge sources [86,87,88]. The sponge *Halichondria rugosa* from South China Sea harboured a *Bacillus pumilus* strain which had lipase activity [86]. From the sponge, *Aplysina aerophoba*, a novel esterase was isolated from a marine *Bacillus* species [87]. Novel halotolerant lipases were found using metagenomic libraries of the marine sponge, *Haliclona simulans* [88]. These successful applications further highlight the potential unique and novel biocatalytic enzymes that could be mined from the marine biome, especially from sponges.

### Mining the Marine Biome for Novel Biocatalysts

The vast majority of microorganisms on the planet are unable to be grown routinely in the laboratory which has been a major challenge when searching for new or novel biocatalysts. It is estimated that 99% of bacteria cannot be cultured by traditional methods [89,90]. Hence, developing optimised culturing conditions and media supplements to culture these organisms has been the target of several research initiatives [91,92]. It is likely that a broad range of media will be required to access the biodiversity that is currently unavailable to us [93]. Counterintuitively, often low nutrient concentrations improve cultivation efficiencies [94,95]. Where culturability cannot be achieved, applying metagenomics to uncover novel enzymes can circumvent these difficulties and hence increase the chance of positive biocatalytic hits from complex samples [96]. 

For this reason, metagenomics has emerged as an important tool in the analysis of microbial communities and for the detection of novel enzymes [97]. Prior to the construction of a metagenomic library, the samples must be stored to maintain genomic (g)DNA integrity. To enhance the DNA recovered, often filter and centrifugation methods are applied to capture as much of the DNA as possible. Filtration was compared to centrifugation and was found to be the most efficient for isolation purposes [98]. To access the gDNA from the sample, the cells must be lysed, while maintaining the integrity of the gDNA. This is a very important step as this may alter the final results [99]. Techniques are available for the optimisation of gDNA quality, one method uses a modified chemical extraction protocol and results in higher yields, lower shearing and increased lysis efficiency from saline environments when compared to other chemical SDS-based techniques [100]. Other techniques employ a combination of mechanical, chemical and enzymatic lysis to optimise the quality and quantity of extractions from the marine environment. Methods using this combinatory approach were assessed compared to a number of different techniques and was proven to be superior to these. This combination technique was referred to as the THSTI method [101]. 

An important factor in the construction of a metagenomic library is the heterologous host used as the chassis organism for expression studies. *E. coli* strains BL21 and K12 are the most common hosts for the construction of metagenomic libraries [102]. Though they are the most common hosts for functional analysis, not all proteins can be expressed in *E. coli*. Only 40% of enzymatic activity is expressed when genes are randomly cloned into *E. coli* [103]. Due in part to difficulties in the recognition of foreign promoters [104]. To address this issue, heterologous sigma factors have been introduced into *E. coli* to improve expression [105]. There are several factors involved in the expression of a protein. A suitable secretion system is required, with some *E. coli* strains having silenced secretion pathways [106]. Other obstacles include the requirement for rare codons and codon usage bias, which together could make expression of proteins problematic [107,108]. The formation of inclusion bodies is another issue faced when trying to successfully produce an expressed protein in a heterologous system [109]. 

Utilising a selection of hosts which are genetically tractable, such as *Pseudomonas, Streptomyces, Bacillus, Agrobacterium* and *Rhodococcus,* will increase the chance of expressing the metagenomic library, thus increasing the likelihood of identifying enzymes of interest [110]. Often using hosts other than *E. coli*, such as hosts that are naturally present in the environment from which the sample was collected, can increase the likelihood of the expression of the genes of interest. The expression of a protein in the native form can be difficult when expressing it in *E. coli*. The expression of the flavoprotein, hydroxylases 3-Hydroxybenzoate 6-Hydroxylase (3HB6H) from *Rhodococcus jostii* RHA1 is problematic as the expressed protein can be bound to two components of the cytoplasmic membrane of *E. coli*, phosphatidylglycerol and phosphatidylethanolamine. The native expression is only bound to phosphatidylinositol. *R. jostii* RHA1#2 was developed as an alternative host to produce the native form of the protein [111]. This could also be seen when soil was screened for β-galactosidase activity. *Sinorhizobium meliloti* was used as a host of the metagenomic library and β-galactosidase activity was successfully identified [112]. A novel esterase was identified using the thermophile, *Thermus thermophilus* as a host in a metagenomic library screen. This study noted that there was a significant increase in the isolation of active clones when *T. thermophilus* was used as a host when compared to *E. coli* as a host [113].

A metagenomics library can be constructed by using either small insert in plasmid vectors or large inserts in fosmids or bacterial artificial chromosomes (BAC). Large insert fosmids and BAC libraries can allow us to study complete metabolic pathways [114]. However small-insert libraries are more useful for the isolation of activities encoded by a single gene or a quantitative study of community composition [115,116]. The pCC1FOS fosmid vector (Epicentre) is commonly used when constructing a metagenomic library with large insert sizes of 35–45 kb [117]. The pCC1FOS fosmid is advantageous over other vectors for a number of reasons including the presence of a chloramphenicol resistance marker gene within the vector, which is superior to other common markers such as ampicillin when constructing libraries [118]. It is a broad-host-range fosmid, being maintained in diverse members of *Proteobacteria* [119]. It is inducible for greater expression rates, with the supplementation of L-arabinose in the media [120]. Recent studies have modified the pCC1FOS backbone to include *oriT,* thus allowing the fosmid to be conjugated into a wide range of species. This modified fosmid is now referred to as pRS44 [121,122].

When screening a metagenomic library, choosing the appropriate assay to detect new activities is also important. For compounds other than hydrolases, there are few high throughput techniques available for the screening of large metagenomic libraries. To detect enzymatic activity of interest, a large number of clones often need to be screened. This makes the development of high-throughput screening methods essential. Important developments in screening methods include the ability for high-throughput screening of hydrolases [123,124,125]. An important industrial example of a hydrolase is sulfatase [126]. One methodology utilises an ultraviolet (UV) light detection system for the high throughput screening of sulfatases, this technology is based on the formation of *N*-methylisoindole by sulfatases [127]. Another new high-throughput method uses colorimetric assays for sulfatase activity [128]. The substrates that the enzymes are screened against are also of great importance. To achieve high hit rates of enzymatic activities, multiple substrates should be incorporated into a screen. This can increase the chances of identifying activity and may also demonstrate activity specificity [129]. Screening multiple substrates for enzyme specificity is important as it can identify new substrates that biocatalysts can convert to more value-added compounds. This is demonstrated in a study whereby flavin-dependent enzymes, specifically vanillyl alcohol oxidase and eugenol oxidase are used to catalyse the oxidation of *para*-substituted phenols [130]. Both these enzymes share sequence, secondary, and tertiary similarities, however they differ in their substrate specificity [131,132]. Screening many substrates can be time consuming however this group developed a high throughput colorimetric screening assay using xylenol orange to rapidly screen twenty-four potential substrates for enzyme specificity. Utilising high-performance thin-layer chromatography (HPTLC) is an example of a rapid method of screening and important flavonoid-modifying enzymes have been identified using this technique [133]. Ultra-high-throughput has emerged as being a promising technique due to its low cost and capacity to screen a large number of clones. Ultra-high-throughput technologies differ to high-throughput methods as these techniques can often screen up to 10 billon potential activities per day. One example consists of a single clone being encapsulated with a specific substrate into a droplet and the protein activity measured based on absorbance or fluorescence [134,135]. 

Biosensors can also be used to detect enzymatic activity. A high-throughput GFP based biosensor was used to identify biocatalysts for the transformation of lignin into commercially important compounds [136]. While whole cell biosensors have been routinely applied in environmental monitoring, their application in the drug synthesis pipeline is an emerging area of technology development [137,138,139]. The ability to monitor substrate to product conversion in situ within a production facility can streamline efficiencies within the pharmaceutical process. Whole cell biosensors are typically comprised of two modules; the signal/analyte responsive protein and the reporter module which produces a measurable output in the form of e.g., fluorescence (*gfp*), luminescence (*lux*), or enzymatic activity (*lacZ*) [140]. As such, this represents a classic modular system with significant potential for development using systems and synthetic biology approaches. The sensory component of biosensors is generally a transcription factor, with LysR-type transcriptional regulators (LTTR) having demonstrated considerable potential in this regard [141,142]. Notwithstanding their potential, limited understanding of the evolutionary drivers of LTTR divergence, and the constraints on inter-domain coupling between heterologous LTTRs, has hampered the development of hybrid ‘specific-purpose’ biosensors [143]. Other classes of biosensors have already been successfully applied in monitoring the production of cis,cis-muconic acid [141]. Furthermore, a real-time highly sensitive microacoustic (Love Wave) device was used for the detection of okadaic acid, specifically using HepG2 cell lines as the primary sensing elements [137]. Biosensors have also been used to screen for amidases from a metagenomics library, in this case utilising a benzoate-responsive biosensor [144]. While many methods of detection rely on solid media screens, a high-throughput liquid state assay has been developed to detect lipase activity from metagenomics libraries [145]. This technique provides reproducibility and more accurate quantification than its solid media based counterpart. 

Apart from metagenomics analysis, the mining of genome sequences has been a useful tool to discover new biocatalysts from a number of environments [146,147]. Currently there are large volumes of genomic information in databases from marine sources and this is mostly due to decreased sequencing cost and more efficient sequencing and annotation [148]. These DNA sequences can be mined for potentially novel bioactives. Mining genome sequence techniques have successfully been applied and have been used to identify fluorinases from *Streptomyces*, *Norcardia*, and *Actinoplanes* species. These fluorinases have 80% to 87% sequence identity to a known fluorinase from *Streptomyces cattleya*. The fluorinase from *Streptomyces* produces the fluorometabolites, fluoroacetate and 4-fluo-rothreonine, similar to *S. cattleya*. However, it is also able to produce a variety of unidentified fluorometabolites that could have industrial importance [149]. Bioinformatic computational methods have been developed in order to characterize unknown proteins. Algorithms investigate predictive active site as targets for computational programmes so that they can predict the active sites of a proteins, thus inferring function [150,151]. To also achieve functional analysis, sequenced-based methods use alignments and 3D structures to predict function [152]. Combinatory techniques which combined the structure predictions and shapes of proteins with sequenced based methodologies are some of the most useful [153]. Nevertheless, there is a large amount of genes for which their function has yet to be described. This functional prediction difficulty is not only limited to rare genes. Many genes that are required for the minimum function of life have yet to be ascribed a purpose. However it has been predicted that many of these will be resolved in the coming years, due to computational advancements [154]. Another recent study found that integrating protein-protein interactions and protein domain information aided in the accuracy of predicting protein function [155]. These protein models can all greatly aid in the discovery of novel biocatalysts from an already existing database of sequences. 

## 3. Industrially Relevant Properties of Novel Marine Lipases/Esterases

Lipase and esterases catalyse the hydrolysis of ester bonds [156]. These enzymes are among the most useful in industry. Therefore, identifying novel lipases and esterases from extreme environments, such as the marine, could supply a variety of industries with more cost effective and environmental friendly processes. Both lipase and esterase activities have been detected in the marine environment. An example of a lipase with industrial importance was isolated from the marine fungus, *Aspergillus awamori* [157]. This lipase could reduce up to 92% of fat in oil-laden effluent. A lipase from a marine *Janibacter* species has important oil modification properties that are applicable in the food industry [158]. Moreover, it is important to note that this lipase is active at low temperatures, which would be a beneficial property that is applicable to the food industry. The lipase, MAS1 from a marine *Streptomyces* also fulfils green chemistry principles by performing an environmentally friendly process, and moreover, uses a waste product, waste cooking oil, to produce commercially valuable biodiesel [159]. 

Employing metagenomic strategies as a tool for discovery of novel biocatalysts provides the ability to identify enzymes from unculturable organisms [160]. Numerous novel enzymes from different environments have been discovered in the last number of years [61]. Marine lipolytic enzymes can show unusual biochemical properties which are highly applicable to industrial processes. Peng and colleagues screened a metagenomic library constructed from marine sediments [161]. They discovered an alkaline-stable lipase which partially hydrolyses milkfat. This is highly valuable to the dairy industry as it develops important organoleptic qualities, especially flavour. Important alkali lipases have also been discovered from a metagenomic library from the marine sponge, *Iricina* [162]. This lipase shows great potential to be utilised in the detergent industry, as well as in organic synthesis processes due to its ability to tolerate organic solvents. Marine sponges are a valuable resource for novel enzymes, however marine sediment has also been screened with great success. A novel thermostable esterase was discovered from sediment with potential applications in the biosynthesis of flavour esters [163]. Metagenomic libraries of samples from Polycyclic Aromatic Hydrocarbon (PAH) and oil contaminated seawater have also been screened and five cold-adapted esterases successfully identified [164]. These esterases are important as they show high activity at low temperatures and were highly resistance to detergents, salts and solvents. A novel esterase, which shows less than 27% sequence identity to the characterised lipolytic enzymes, was found by screening a metagenomic library from samples from the South China Sea. This enzyme can be particularly useful for industrial processes due to its high activity and ability to function at very high salt concentrations [165]. While many important and useful enzymes have been found in the marine environment, there is still a large reservoir of enzymes yet to be discovered.

## 4. Identification of Novel Enzymes from the Marine Environment by Metagenomic Analysis—A Proof of Concept Case Study

**Figure 4:**
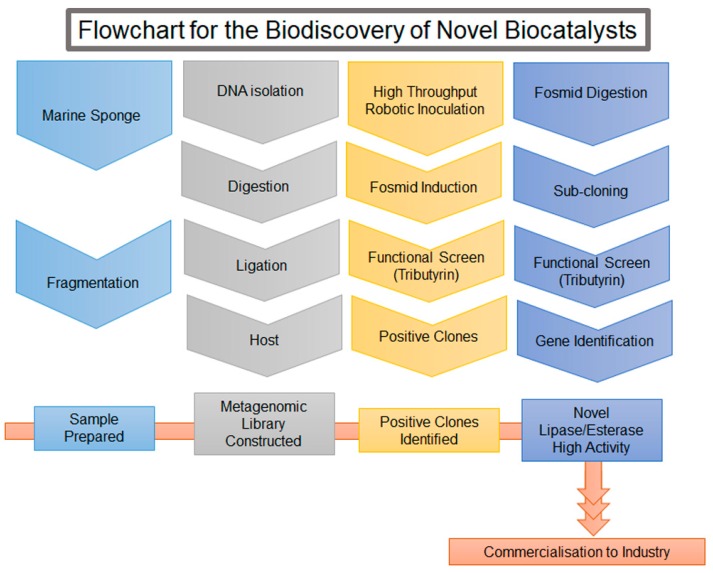
Flowchart for the Biodiscovery of Novel Biocatalysts.

To screen large quantities of enzymes of interest, metagenomic libraries coupled with screening technologies is currently the method of choice. In order to construct a metagenomic library the first step is to harvest the environmental sample and carry out the isolation of the DNA. To detect lipase/esterase activity, a metagenomic library was screened. In our case study, sponge samples were collected from Lough Hyne, a marine lake in the Southwest of Ireland. A metagenomics library (20,352 clones) was constructed from the marine sponge *Axinella dissimilis* using one of the common hosts, *E. coli* EPI300 and the fosmid vector pCCFOS1 [166]. The entire process that we applied in the case study is outlined in Figure 4. Positive clones were detected by the appearance of a clear halo surrounding the colony indicating the hydrolysis of tributyrin to glycerol and butyric acid [156]. To screen the large library a QBIXS2-XT Microbial Colony Picker robot was employed to automate a rapid high throughput screen. This screen is based on the ability of both lipase and esterase enzymes to metabolise tributyrin. Further analysis is usually needed to distinguish the catalytic range of the positive clones. To induce the fosmid from a single copy to a high copy number, 0.01% (*w*/*v*) L-arabinose was added to the media. This can increase the levels of expressed protein thus increasing the likelihood of detecting a positive clone. From these lipase/esterase positive clones, the fosmid was extracted and digested by the BamHI restriction enzyme. The digested fragments were sub-cloned into the expression vector, pET28a(+) and conjugated into *E. coli* BL21 (DE3). The enzymes that were discovered were subsequently characterised for specificity and speed of reaction [167,168]. Two interesting and novel lipase/esterases were found by screening the marine sponge *Axinella dissimilis* metagenomic library. Each of these hydrolases showed unique uncommon properties. The first hydrolase discovered has the ability to hydrolyse a challenging substrate with a remote stereocenter and gives a very high yield of specific products. The other hydrolase discovered in the metagenomics library showed an unusually rapid reaction time with the target substrate metabolising tributyrin. These novel lipases/esterases display a unique and promising activity. However, further improvements in the capabilities of these enzymes could be accomplished by directed evolution strategies thus increasing their value for industrial application. [169].

Outlined are the steps involved in the discovery of novel enzymes. The process begins with sample collection and preparation, which involved the collection of a marine sponge followed by fragmentation. The metagenomic library was constructed from the isolated DNA, which was restricted and ligated into a fosmid and inserted into a host. The library was screened and positive fosmid clones identified. The fosmid was subsequently sub-cloned into an expression plasmid and screened for activity again. The genes of interest were sequenced and found to have low sequence identity with other lipases/esterases, thus identifying our genes as novel. Several steps needed to be completed to use the enzyme in a commercial setting, these include biocharacterisation, condition optimisation and process validation. 

## 5. Advantages and Regulatory Compliance of Green Chemistry Solutions

Green chemistry offers many solutions to current pollution problems. It is also a field rapidly increasing in market value. The use of enzymes in industrial processes can increase production and lower hazardous outputs. Enzymes have replaced chemical processes in several industries including the food, beverage, textile, detergent, and pharmaceutical industries. Biocatalysts are becoming more profitable and replacing some traditional environmentally challenging processes in industry. The use of enzymes is now becoming inevitable due to resource limitation and increasing demands. Much work has been accomplished by the Environmental Protection Agency (EPA) and the European Union, to monitor and evaluate the toxicity of commonly used chemicals, through the Toxic Substances Control Act (TSCA) and Registration, Evaluation, Authorization and Restriction of Chemicals (REACH) Act [170,171]. Through the application of more biocatalysts, much of the highly toxic chemicals monitored and highlighted by these agencies will no longer be necessary. Applying green chemistry solutions would allow for ease of compliance with the various plethora of regulations.

The marine environment is a relatively unexplored resource of enzymes. Industrial processes need to be conducted under a wide range of conditions, many of these conditions are similar to those found in the marine environment, such as in curing in the food industry. Since the majority of microorganisms are not culturable routinely under laboratory conditions, investigating the needs for the growth of these organism is pertinent, however heterologous expression often solves this issue. Metagenomic strategies still currently offer one of the best opportunities to uncover new bioactives. While utilising *E. coli* as the library host is still the most favoured, other hosts should also be considered for expression analysis in particular those from the environment that the protein is natively expressed in. High-throughput screening technologies of large metagenomic libraries have increased the chances of the biodiscovery of biocatalysts. All of these methodologies lead to more accurate and faster discovery of new types of enzymes. We have successfully demonstrated the application of metagenomics to identify industrially important enzymes that adhere to green chemistry principles. With the application of these enzymes and more biocatalysts to be discovered, the future of the “Green Chemistry Landscape” looks very promising.

## Figures and Tables

**Table 1:** Definitions of concepts involved in green metrics.

Concept	Abbreviation	Definition
Atom Economy	AE	The amount of raw material used as a substrate that becomes a useful product with the minimum waste production
Environmental Quotient	EQ	The relation between the mass of waste generated during a process and the environmental impact that the waste causes
Reaction Mass Efficiency	RME	This concept takes into consideration not only the atom economy but also the yield and stoichiometry of a chemical reaction
Mass Intensity	MI	The total mass of material, such as reactants, solvents, and reagents, used to produce a specific mass of product
The Life-Cycle Assessment	LCA	A technique to evaluate the environmental impact of the entire life of a product, this included all the steps from the extraction of raw material, manufacturing, storing, distribution, use, disposal and recycling

**Table 2:** Important extremozymes and their industrially important properties.

Extremozyme	Source	Microorganism	Property
Lipase	Waters of Baek-du mountain	*Acinetobacter baumannii*	High activity at low temperatures [72]
	China	*Yersinia enterocolitica*	Activity over a broad range of temperatures (0–60 °C) [73]
	Saline soil from China	*Oceanobacillus rekensis*	80% of activity at 10 °C and fairly active in the presence of long-chain alcohols [74]
	Antarctica	*Bacillus pumilus*	80% of their activity at 10 °C [75]
	Intertidal flat of the Yellow Sea in Korea	*Photobacterium lipolyticum*	Activity maintained at 5 °C [76]
Protease	Indian ocean	*Marinobacter*	60% activity is maintained at 80 °C [77]
	Wastewater	*Bacillus licheniformis*	Optimum temperature at 70 °C and stability towards nonionic and anionic surfactants [78]
α-Amylase	Antarctic ice-shell	*Pseudoalteromonas haloplanktis*	80% of enzyme activity at high NaCl concentrations [79]
	Wastewater	*Bacillus licheniformis*	Optimum temperature at 90 °C and stability towards nonionic and anionic surfactants [78]
